# A Simple, Reliable, and Inexpensive Solution for Contact Color Measurement in Small Plant Samples

**DOI:** 10.3390/s20082348

**Published:** 2020-04-20

**Authors:** Patricia Sanmartín, Michela Gambino, Elsa Fuentes, Miguel Serrano

**Affiliations:** 1 Departamento de Edafoloxía e Química Agrícola, Facultade de Farmacia, Universidade de Santiago de Compostela, 15782 Santiago de Compostela, Spain; elsa.fuentes.alonso@usc.es; 2 Department of Veterinary and Animal Sciences, University of Copenhagen, Stigbøjlen 4, 1870 Frederiksberg, Denmark; mgambino@sund.ku.dk; 3 Department of Botany, Faculty of Pharmacy, University of Santiago de Compostela, 15782 Santiago de Compostela, Galiza, Spain

**Keywords:** Campanulaceae, CIELAB color system, color characterization, nondestructive assessment, reflectance colorimetry, templates

## Abstract

Correct color measurement by contact-type color measuring devices requires that the sample surface fully covers the head of the device, so their use on small samples remains a challenge. Here, we propose to use cardboard adaptors on the two aperture masks (3 and 8 mm diameter measuring area) of a broadly used portable spectrophotometer. Adaptors in black and white to reduce the measuring area by 50% and 70% were applied in this study. Representatives of the family Campanulaceae have been used to test the methodology, given the occurrence of small leaves. Our results show that, following colorimetric criteria, the only setting providing indistinguishable colors according to the perception of the human eye is the use of a 50%-reducing adaptor on the 3-mm aperture. In addition, statistical analysis suggests the use of the white adaptor. Our contribution offers a sound measurement technique to gather ecological information from the color of leaves, petals, and other small samples.

## 1. Introduction

The color of plant organs, particularly of leaves and flowers, is a phenotypic trait traditionally used as a visual indicator of the plant physiological status. Indeed, environmental stress regarding light quantity and quality, nutrients, temperature, and drought result in a change of plant color [1]. These correlations are so informative that color is one of the phenotypic traits for monitoring plant growth in high-throughput classification approaches [2]. The color of leaves and flowers fulfill also an ecological role in plants, since bright colors attract pollinators and seed dispersers [3]. It has also been hypothesized that insects could interpret the color intensity of plant leaves as a signal of the strength of the plant, thus being attracted or repelled [4,5]. Leaves color reflects also the accumulation of secondary metabolites with important chromatic components, which can have a great commercial and therapeutic value. For example, the purple color of bud and leaves in tea cultivars (e.g., Benibana-cha, Sunrouge tea, Zijuan tea, and Ziyan tea) is associated with the accumulation of anthocyanins, which have been shown to have a therapeutic role against colorectal carcinoma cells [6] and in reinforcing the brain’s antioxidant capacity in mice [7]. In addition, since these metabolites accumulate in response to the plant interaction with soil, the color of leaves and petals is an indicator of the characteristics of the soil. Well known examples are the overexpression of carotenoids and anthocyanins in plants growing in soil with a high salt concentration [8] or the changes in color of flowers in Hortensia (*Hydrangea macrophylla*), which varies from blue to pink, according to the soil acidity. Moreover, color transitions and pigment variation in plants also provide information pertaining to the genetic variability within and among populations and taxa, allowing the formulation of evolutionary hypotheses in a phylogenetic framework [9,10]. Changes in color, both in vegetative and reproductive organs [11], evince important evolutionary processes like pollinator-mediated reproductive isolation [12]. Finally, plant disease, including fungal and bacterial parasites, cause lesions and chromatic changes of leaves and flowers [13,14]. Their early perception is fundamental to act timely to eradicate the disease and to limit economic loss, or to monitor the interaction of plants with damaging organisms or environmental factors. Given the intimate connection of flower and leaf color with the physiology of the plant, its quantification by the human eye has been traditionally used to guide breeding experiments to accumulate metabolites, but also as a proxy for soil parameters and the detection of disease.

Unfortunately, color detection and classification by the human eye are extremely unreliable, because of their dependency from the experience and the capacity of the observer. The choice of a color by a living organism is based on psychological and evolutionary or survival criteria. For example, among the colors distinguished by the human eye, green is perceived more readily and with the largest shift in their hue than any other color, because of the combined perception of rods and cones [15]. It is hypothesized that this could represent an adaptation to the environment of primates in search of food [16].

Color can be described in an objective and precise way through the use of spectrophotometers, colorimeters, or chroma-meters that describe the reflected color in the standardized CIELAB color system (see e.g., [17,18,19]). They work by contact on a surface, like other contact-type spectrometer devices [20], and they average the light reflected from an aperture on the head of the device. The aperture is circular and has a diameter between 3 and 60 mm (see e.g., [18,19,21]). Surfaces with an area in a different shape or smaller than the aperture of the device do not reflect entirely the emitted light, thus leading to a consistent loss of light and to unreliable results. To perform the color measurement correctly, the sample surface must cover entirely the aperture of the device to prevent the leakage of light or interference from external light. For this reason, the color cannot be measured when the target area is smaller than 3 mm and/or not circular.

To address this issue, we propose to reduce by 50% and 70% the aperture area of a portable spectrophotometer with cardboard adaptors in white and black colors, in order to describe the color in the CIELAB space of small biological samples, such as leaves and petals. Thus, we further enhance the usability of the sensor device by extending its application to smaller samples than those for which it was designed. This unlocks previously unexplored ways to tackle current issues in the environmental monitoring field, such as the biodiversity response to climate change. In this frame, we measured the color of leaves from species of the genus of *Jasione* and others of the Campanulaceae family, thus validating this methodology within a broad phylogenetic group with species of different ecology and frequently with small-sized leaves [22]. This group encompasses a number of alpine species, with a habit of dense rosettes with small leaves, this being a characteristic plant adaptation to alpine environments [23]. In our work, the use of leaves big enough to be measured with the large colorimeter aperture also guaranteed the reliability of this methodology for leaves where only the smallest aperture of the colorimeter aperture can be used, as is the case of many the alpine species. In addition, we included herbarium samples, where dry plant tissues are conserved as valuable ecological, systematic and historical evidences of plant biodiversity. By extending our methodology to conserved dry samples, we propose the use of colorimetry as a tool to describe the color of leaves and petals after some time and monitoring the conservation conditions of these specimens.

## 2. Materials and Methods

### 2.1. Plant Materials

The genus *Jasione* has many representatives occurring in mountain environments [24], having spread around the Mediterranean basin with a number of species endemic of the alpine ranges of the region [25]. They belong to the alpine flora: the mountain zone located above tree line and below the permanent snow line and considered a conservation target [26]. Alpine plant communities constitute some of the most threatened ecosystems in the world by climate change [27]. Thus, alpine flora are currently the subject of a number of international monitoring projects to study the response of plants to global temperature increases and other environmental stressors [28]. Color variation in the leaves of *Jasione* could be used as an ecological indicator in future monitoring studies on alpine plant species. Nevertheless, the peculiar growth of many alpine plant taxa, forming a cushion of dense rosettes of small leaves as a common evolutionary adaptation [23], represents a challenge for the use of color as a proxy of physiological status.

**Figure 1:**
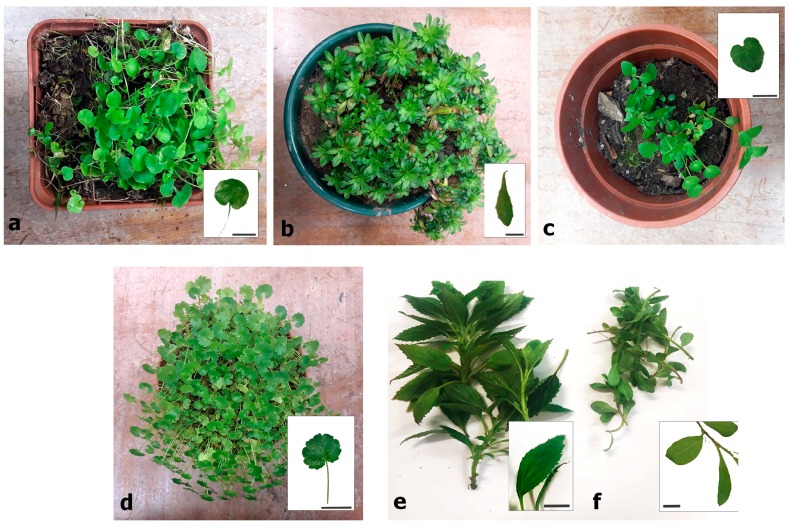
Pictures of fresh plant specimens used in the study. From left to right and top to bottom: (**a**) *Hesperocodon hederaceus* (L.) Eddie & Cupido, (**b**) *Jasione laevis* Lam, (**c**) *Campanula rotundifolia* L., (**d**) *Campanula isophylla* Moretti, (**e**) *Trachelium caeruleum* L., and, (**f**) *Jasione montana* L. Scale bars are 2 cm.

**Figure 2:**
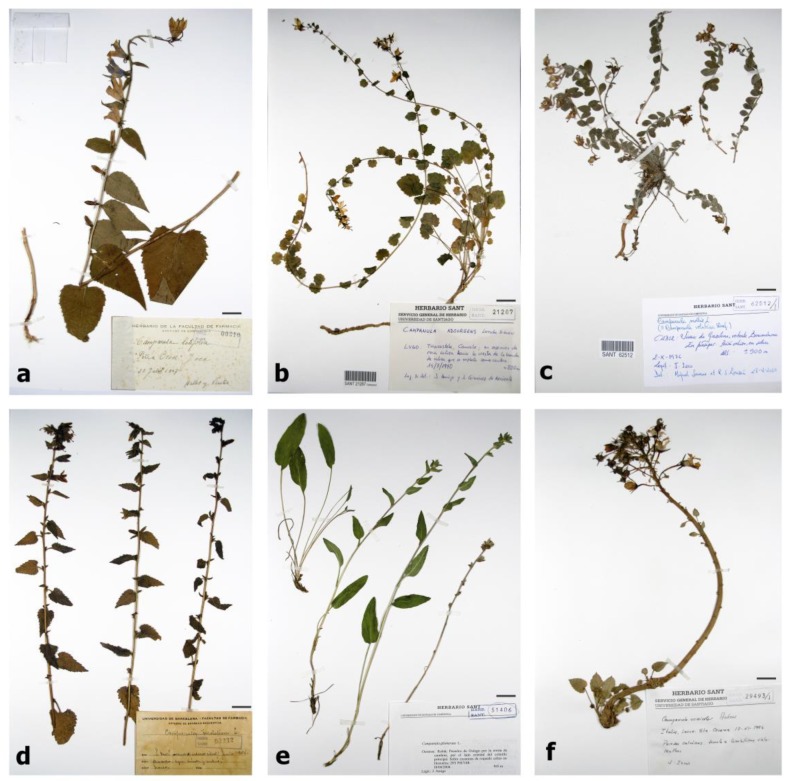
Pictures of herbarium plant specimens used in the study. From left to right and top to bottom: (**a**) *Campanula latifolia* L., (**b**) *Campanula adsurgens* Levier & Leresche, (**c**) *Campanula mollis* L., (**d**) *Campanula trachelium* L., (**e**) *Campanula glomerata* L., and (**f**) *Campanula versicolor* Andrews. Scale bars are 2 cm.

For these reasons, the proposed methodology has been tested on six fresh plants (*Jasione montana*, L. *Trachelium caeruleum* L., *Campanula rotundifolia* L., *Campanula isophylla* Moretti, *Hesperodocon hederaceus* (L.) Eddie & Cupido., and *Jasione laevis Lam*, Figure 1) and six plants from herbarium (*Campanula adsurgens* Levier & Leresche, *Campanula latifolia* L., *Campanula mollis* L., *Campanula glomerata* L., *Campanula trachelium* L., and *Campanula versicolor* Andrews, Figure 2), all belonging to the family of Campanulaceae. The two species of *Jasione* were selected from populations with wide leaves in order to satisfy the conditions required by the study (i.e., 11 mm of diameter in MAV without adaptors, see Section 2.2.). These plants belong to populations of *Jasione laevis* subsp. laevis and *Jasione montana* var. *latifolia* Pugsley.

The herbarium record of the University of Santiago de Compostela, Spain (SANT) was accessed to obtain the specimens. Each herbarium specimen was checked for possible misidentification, and the herbarium code, name of the species, sampling location, collector, and year were recorded (Table 1).

### 2.2. Color Measurements with and Without the Adaptors

**Figure 3:**
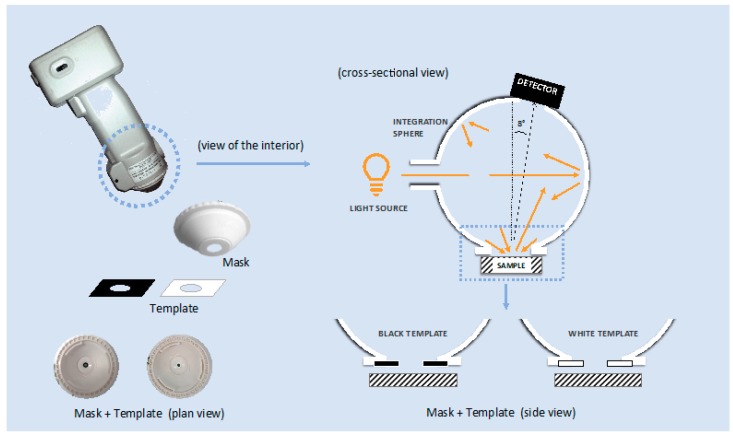
Schematic diagram showing the custom-designed set up for color measurement provided by, the portable spectrophotometer. The template partially plugs the sample port that becomes 50% or 70% smaller and, in turn, expanding the sphere wall.

The color was measured with a portable spectrophotometer Konica Minolta CM-700d (KONICA MINOLTA, INC., Tokyo, Japan), a contact-type color measuring device commonly used in scientific works. A spectrophotometer is a specific type of spectrometer designed to measure light over the visible and near-visible portion of the electromagnetic spectrum, i.e., from 360 to 740 nm. It uses an optic set up of diffuse illumination geometry (recommended by the CIE, “International Commission on Illumination”) accomplished by an integrating sphere covered with a white reflective coating and with 8°/diffuse illumination/viewing geometry (Figure 3). The sphere acts both as a means of producing a diffuse light source through a series of reflections off the white sphere wall, and for the collection of light from the sample surface, placed in the entrance or sample port. The spectrophotometric method utilizes multiple sensors to measure the spectral reflectance of the sample. The sensor’s electronics then calculate the tristimulus values from the spectral reflectance data using integration. The device is equipped with the CM-S100w (SpectraMagicTM NX) software (KONICA MINOLTA, INC., Tokyo, Japan).

**Figure 4:**
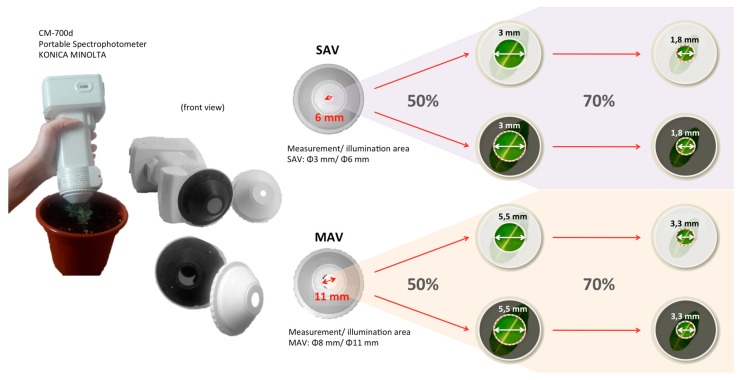
Explanatory diagram of the reduction of the measurement area of the device by means of adaptors in black and white.

Measurements were made in the specular component included (SCI) mode, in which the gloss trap of the spectrophotometer is closed, includes the total reflectance (considering both specular and diffuse reflections), with illuminant D65, observer 2°, and both circular aperture masks: an aperture for medium area view (MAV) with a diameter of 11 mm, measuring a circular area of 8 mm of diameter, and an aperture for small area view (SAV) with a diameter of 6 mm, measuring a circular area of 3 mm of diameter. In addition, eight types of adaptor, four for each aperture mask, have been designed and realized in cardboard, in black or white color, and decreasing the tested surface by 50% (hereinafter called “black 50%” and “white 50%”, respectively) or 70% (hereinafter called “black 70%” and “white 70%”, respectively) (Figure 4). By employing the adaptors, the sample port opening (hole) is reduced to 50% or to 70% by the template, which expand the sphere wall (Figure 3 and Figure 4).

Color measurements were analyzed by considering the CIELAB color system [17], the most widely accepted by both industry and scientific community. It represents each color by means of three scalar parameters or the Cartesian coordinates: L*, lightness or luminosity of color, which varies from 0 (absolute black) to 100 (absolute white), a*, associated with changes in redness- greenness (positive a* is red and negative a* is green), and b*, associated with changes in yellowness-blueness (positive b* is yellow and negative b* is blue).

Five healthy leaves from each species of Campanulaceae were randomly picked and, on the surface of each of them, five points were randomly selected to measure the color, following previous works [29,30]. Although the aim of the work is not to define the species color variability, the number of small leaves and of measurements in each leaf was high enough to average the color variation in small leaves of the Campanulaceae species, in agreement with previous works that analyzed cumulative averages for parameters L*, a*, and b* on heterogeneous surfaces [21,29,30]. Five different set-ups for each of the two aperture masks, MAV and SAV, were used to perform the color record: no adaptor, black 50%, white 50%, black 70%, and white 70%. The color values obtained from the reduced area have been compared with the recorded color without adaptor, using colorimetric criteria and statistical tools.

Because the inhomogeneous pattern of the vein network, the convexity of the leaf surface, and the intrinsic features of each leaf in each plant may affect the determination of color, it should be established if the new method developed and here proposed can be extended to other plant samples. In order to address this concern, four patches homogenously colored in green (green, foliage, yellow green, and bluish green) from the color-checker color rendition chart with 24 color patches by Gretag Macbeth [31,32] were measured with the same protocol used for the leaves, as described in the previous paragraph.

### 2.3. Colorimetric Analysis

Partial (∆L*, ∆a*, ∆b*) and total (∆E*_ab_) color differences have been calculated in absolute values with the following Equations (1)–(4):

∆L* = L*_i_ − L*_x_ (1)

∆a* = a*_i_ − a*_x_ (2)

∆b* = b*_i_ − b*_x_ (3)

∆E*_ab_ = [(∆L*)^2^ + (∆a*)^2^ + (∆b*)^2^]^1/2^ (4)

where L*_i_, a*_i_, and b*_i_ are the color parameters by using the adaptor and L*_x_, a*_x_, and b*_x_ are the same parameters by using no adaptor. Here, the threshold of 3 CIELAB units has been adopted as the limit of rigorous color tolerance or noticeable by an observer with normal color vision, as in several other studies [32,33,34,35,36,37,38].

### 2.4. Statistical Analysis

For a robust evaluation of the effect of the adaptors on the color measurements, the collected data have been analyzed with three different multivariate approaches: Principal Coordinates Analysis (PCoA), quadratic discriminant analysis of multiple groups (QDA), and a combined approach based on k-means and agglomerative clustering analyses. PCoA was also applied to the measurements obtained from the four patches (green, foliage, yellow green, and bluish green) of the Gretag Macbeth color-checker color rendition chart.

With the PCoA analysis the contribution from each adaptor type (black or white, 70% or 50% reduction, or no adaptor) on both MAV and SAV to the colorimetric information (L*, a*, and b*) was assessed. For each species, data were standardized by subtracting the mean of measurements with no adaptor to preserve the units of deviation from the actual color and layout of all measurements in the same multivariate space. The measurements were distributed in a 2-dimensional Euclidean ordination space using ggplot2 and Stat R packages [39].

To assess the similarity of the measurements obtained with and without the different tested adaptors, a QDA was performed. The individual measurements were assigned to predefined or actual groups (SAV and MAV with different adaptors or without adaptors), and the adaptor type most like SAV and MAV (i.e., those whose observations are significantly misclassified as observations obtained from SAV and MAV with no adaptors) were quantitatively identified from the results of the QDA confusion matrix. The analyses were run separately for SAV and MAV measurements and, in order to compare all species together, data were standardized by subtracting the mean of measurements, respectively from SAV or MAV with no adaptor. A leave-out-out cross-validation was performed after the analysis to validate the QDA. Multivariate normality was assessed with the MVN R package [40] and the MASS R package was used for the remaining analysis [41].

To verify the results pointed out by the QDA, an approach combining k-means and agglomerative clustering was performed on the same data from SAV, treated as for QDA. By k-means cluster analysis, similar color measurements were categorized by defining clusters to minimize the total intra-cluster variation. The optimal number of k-clusters in each species was determined using 30 indices provided by the R package NbClust [42]. The agglomerative clustering was conducted by computing a dissimilarity matrix based on Euclidean distances and the Ward.D2 agglomeration method in the Stats R package to produce a hierarchical tree for each species [43]. The identified k-means groups were then superimposed on the dendrogram and, by means of the pvclust R package [44], the statistical support of all clusters was calculated with approximately unbiased (AU) *p*-values by multiscaling bootstrap resampling. Clusters with an AU *p*-value equal to or greater than 95% were considered to be strongly supported by data.

## 3. Results

A colorimetric analysis of the gathered measurement from each species of Campanulaceae, as well as the green patches from the Gretag Macbeth color-checker color rendition chart, was performed. Partial (∆L*, ∆a*, ∆b*) and total (∆E*_ab_) color differences using one of the four adaptors and no adaptor were calculated. The latter are summarized in Table 2. Partial differences appear in Table A1. The color parameters most widely varying with the use of the adaptors in plant samples were L* (lightness of color) and b* (associated with changes in yellowness-blueness) as seen in Table A1. L* varied to a larger extent with the use of the white adaptor, increasing the lightness in the registered color (data not shown), and a similar observation can be made for the parameter b*: when the black adaptor is used, the blue component increases in the registered color (data not shown). As expected, widening the diameter of the adaptor from 50% to 70% increases the differences in color calculated with respect to the measurement without adaptor (Table 2). In the green patches similar results were obtained. Furthermore, for the green patch, the parameter a* (associated with changes in redness-greenness) changed the most, closely followed by b*. For the foliage and yellow green patches, L* and b* varied the most, and for the bluish green patch, it was L* and a* to be affected, while the change in b* was practically negligible.

As in other studies, we adopted the threshold of 3 CIELAB units as limit noticeable by an observer with normal color vision (see Section 2.3). With the SAV aperture and the 50% white or black adaptors, none of the twelve studied plant species or of the four Gretag Macbeth patches crossed that threshold in the total difference of color (∆E*_ab_), 2.9 CIELAB units being the highest difference observed (Table 2). Remarkably, the 50% white adaptor on the SAV aperture performed slightly better than the black adaptor on specific plant samples (Table 2). With all other settings, instead, the differences of color spanned from 3.8 to 48.3 CIELAB units (mostly between 20 and 30 CIELAB units) in plant samples and from 6.6 to 57.2 CIELAB units in Gretag Macbeth patches, corresponding to a very noticeable difference with respect to the color measured without adaptor. In the case of plant samples, the impact on color measurements is extreme if the white 50% adaptor is used on the MAV aperture. In the homogeneous patches (except in bluish green), this occurs when employing the white 70% adaptor on the MAV aperture, and to a minor extent on the SAV aperture. The adaptors did not perform better on fresh plants than on their counterpart from the herbarium. It was on two fresh plants, *C. isophylla* and *H. hederaceus*, that measurements with adaptors not only lay within the non-noticeable color differences (<3 CIELAB units), but they even come close to 1 CIELAB unit.

**Figure 5:**
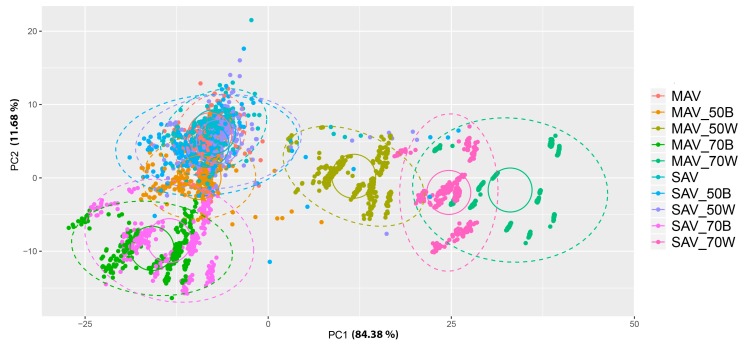
PCoA representing all the color measurements of the plants with and without adaptor. Ellipses around each type of adaptor indicate the dispersion of the measurements and circumferences encircle the centroids of the distributions of measurements of each adaptor type. Samples were colored according to the used adaptor. MAV, medium area view; SAV, small area view; 50B, 50% reduction with black adaptor; 50W, 50% reduction with white adaptor; 70B, 70% reduction with black adaptor; 70W, 70% reduction with white adaptor.

**Figure 6:**
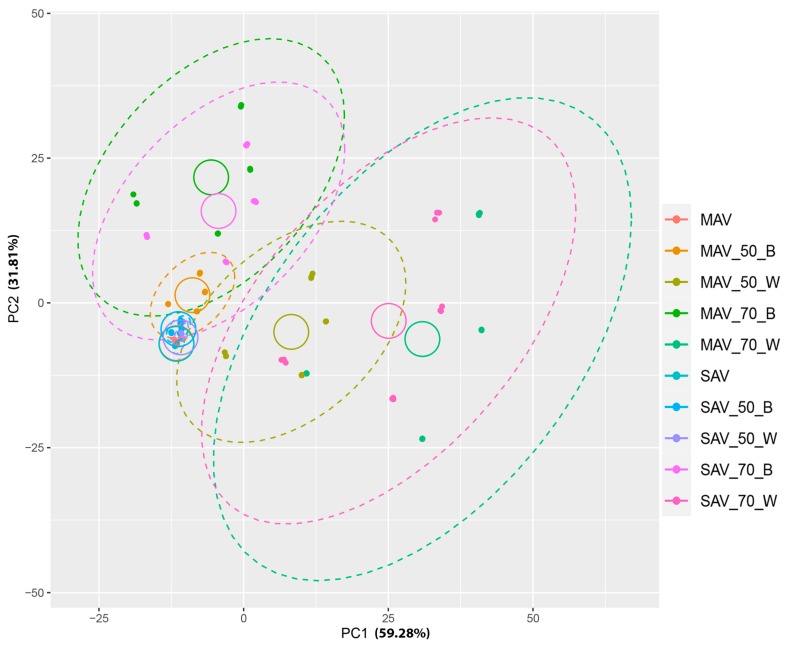
PCoA representing all the color measurements of green patches of the Gretag Macbeth chart with and without adaptor. Ellipses around each type of adaptor indicate the dispersion of the measurements and circumferences encircle the centroids of the distributions of measurements of each adaptor type. Samples were colored according to the used adaptor. MAV, medium area view; SAV, small area view; 50B, 50% reduction with black adaptor; 50W, 50% reduction with white adaptor; 70B, 70% reduction with black adaptor; 70W, 70% reduction with white adaptor.

The contribution from the adaptor type (black or white, 70% or 50%, or no adaptor) on both MAV and SAV to the colorimetric information (L*, a* and b*) was analysed by distributing the measurements in a 2-dimensional Euclidean ordination space with a Principal Coordinates Analysis (PCoA). The color measurements obtained with an adaptor (black or white) reducing 50% of the SAV opening overlaps accurately with the measurements without adaptors. The first two axes explain the major part of the data variability, both in plants (84.38% PC1, 11.68% PC2) (Figure 5) and in the four green Gretag Macbeth patches (59.28 % PC1, 31.81% PC2) (Figure 6). Between the two, the 50% white shows a slightly more accurate approximation than black 50%, as visible from the distance centroids-ellipse. In the case of the MAV, the best adaptor is the black 50%, but overall, it is evident that the measurements obtained by using the remaining adaptors are very far from the real color.

**Figure 7:**
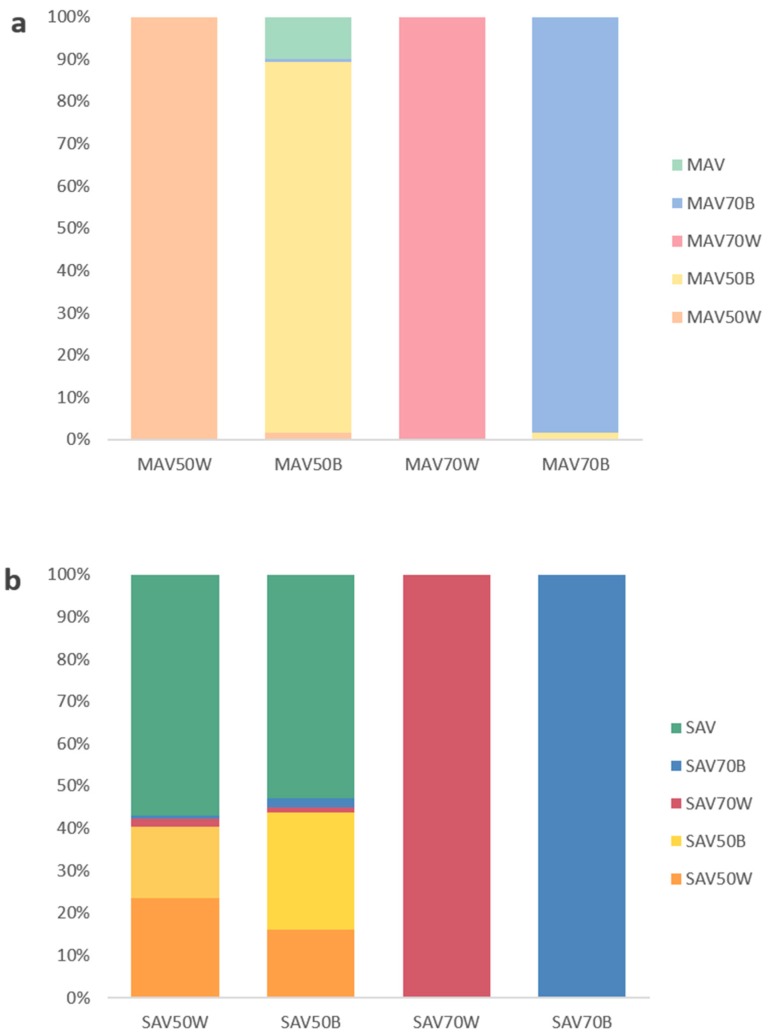
Percentage of the measurements that have been assigned to a predicted group after the use of the adaptors on (**a**) MAV and (**b**) SAV. Samples were colored according to the used adaptor. MAV, medium area view; SAV, small area view; 50B, 50% reduction with black adaptor; 50W, 50% reduction with white adaptor; 70B, 70% reduction with black adaptor; 70W, 70% reduction with white adaptor.

To assess the similarity of the measurements obtained with and without the different adaptors, a quadratic discriminant analysis (QDA) was performed. The mean squared error (MSE) was 0.338 in the SAV analysis and 0.046 in the MAV analysis. The cross-validation of QDA classifications left misclassification rates almost identical to the original analyses, with 0.317 and 0.047 MSE in SAV and MAV, respectively, validating the model in both analyses. The data obtained with SAV reveal a relatively high misclassification rate (33.8 %) in the confusion matrix that can be used to identify the adaptors with a minor impact on the real color measured with SAV. The major part of measurements from SAV with white (57%) and black (53%) 50% adaptors are better classified as obtained with SAV without adaptor than within their predefined group. Thus, to measure the color with these adaptors is as reliable as to measure it without (Figure 7). In addition, some mutual misclassification is detected between the measurements obtained with white and black 50% reducing adaptors: 17% of the measurements obtained with the white adaptor are classified as obtained with the black and 16% of the ones obtained with the black are classified as obtained with the white. Very few measurements from the 50% reducing adaptors were assigned to the 70% reducing adaptors (2%), and no measurement from white or black 70% adaptors was misclassified, indicating that measurements from these adaptors are very dissimilar to those obtained without adaptor. In contrast to SAV, the misclassification rate in the QDA of the data collected with MAV was very low (0.046%). The only significant misclassification in the analysis, showing some similarity between measurements obtained with any adaptor and without adaptor, also explains most of the total misclassification related to MAV: 10% of data collected with the black 50% reducing adaptor to the group of MAV without adaptor. Nevertheless, the percentage obtained with MAV is far from the values obtained with the SAV 50% reducing adaptor, thus suggesting it as the most appropriate adaptor for measuring color in small biological samples.

An approach combining k-means and agglomerative clustering was performed on the data from SAV only, as MAV adaptors has been repeatedly identified as highly dissimilar to the color measured without adaptor by the previous analyses. Table 3 summarizes the clustering of measurements, with the number of k-clusters and the AU p-values of each k-clusters. Except for few data from *C. glomerata* (4% of SAV 50% black and 4% of SAV 50% white) and *C. latifolia* (8% of SAV 50% black), all the measurements obtained with the 50% adaptors fall in the same clusters as with no adaptor. Thus, this approach strongly supports the results of the previous analyses, by establishing that the 50% adaptor is the best solution to obtain a reliable measurement on small samples. Between the two 50% adaptors, the only observable difference is in the case of *C. latifolia*, where the white adaptor performs better than the black.

## 4. Discussion

Trait-based approaches are increasingly widespread due to the important role of color. The use of spectrophotometers, colorimeters, or chroma-meters to measure the color of leaves and flowers as a proxy is a valuable strategy to gather information about the eco-physiological status of plants and the expressed genetic variability within and among populations or taxa. The former is particularly relevant in a world affected by global warming to detect anomalies in crops due to environmental stressors, nutrient deficiencies, contaminants or diseases [45]. In particular, the color of leaves is considered as a key feature for the plant functional traits, since leaves are primary drivers of the capture of light and photosynthesis processes [46]. For instance, the called “winter-red-leaf syndrome” [47,48] has been correlated with a nitrogen deficiency, low carboxylation efficiency and high risk of photoinhibition.

By using a portable version of these devices, color can be measured on-site, thus preserving the integrity of the plant and protecting species that may be at risk of extinction [49]. In addition, a portable device can be useful for botanists, ecophysiologists or ecologists to save specimens for future analysis and compare the color after periods of time [50]. Regular color measurement can be also planned from time to time to monitor the conservation conditions of herbaria: the correct temperature, humidity and light should be constantly maintained to avoid degradation and the specimens’ color is highly indicative of their conservation state [51].

For this study, we chose leaves from 12 different species of the Campanulaceae family, coming from different habitat types and conservation conditions, from living plants to herbarium specimens, and therefore encompassing a wide range of color variations. They belong to a group of plants with a number of representatives from threatened alpine environments. Within them, *Jasione* is a genus in evolutionary relation with numerous endemics of small area, many from alpine habitats, and different edaphic restrictions and ecological preferences [52,53], which makes it suitable to test plant response to edaphic stress. Indeed, plants from alpine ecosystems are also ideal indicators of environmental variations related to global climate change (GLORIA Coordination). Despite their importance, the small size of the leaves gathered in rosettes of many alpine plants [23] offered many challenges to the researchers. Leaves of Campanulaceae though are not the only ones offering a similar challenge.

To perform unbiased free detection of color measurement of small plant leaves, a simple method based on narrowing the field of view of the device is outlined and proposed here. To assess the reliability of the method development outputs, both the perception limit of the human eye (colorimetric criteria) and multivariate statistic approaches have been chosen. It has been demonstrated that 3 mm aperture with an adaptor reducing 50% of the area allows a sound color assessment. In addition, cardboard adaptors in white performed better than their counterpart in black. This conclusion is reached both in the samples of plant leaves and in the homogeneously colored Gretag Macbeth patches, used to test the method for comparative purposes. Since we proved that the method works for both sample types, it is possible to extend it to other types of plant samples, regardless of their specific characteristics (e.g., concavity, intrinsic colorimetric heterogeneity, small defects).

Furthermore, it seems to indicate that the effectiveness of the proposed method is independent of the sample surface texture, defined as the visual characteristic and tactile quality of the surface of a material. This may be largely due to having included the specular component in the measurements (i.e., SCI mode). While the specular component excluded (SCE) mode magnifies the color differences due to the surface of the material, the SCI mode is adequate for analyzing the intrinsic color of objects and minimizes the color differences caused by the texture or surface finish [36].

On the other hand, the better performance of the white template could be related to the optic set up of diffuse illumination geometry, accomplished by an integrating spherical device coated with a white material such as barium sulphate (see Section 2.2). The white adaptor may form a continuum with the sphere, with a material as white as the sphere, thus amplifying the effect of the sphere and providing accurate measurements on SAV.

Also, there are results showing a different behavior of the color parameters (L*, a*, or b*) when recovered with the use of the templates, varying widely both within and among the plant species and color patches. This difference in performance seems to follow a clear pattern: the parameters with a higher value (stronger component color) are those most influenced by the templates. For example, for a bluish green patch, with achromatic values in the yellow-blue component around 1 CIELAB unit (data not shown), the impact on b* parameter is practically negligible when the adapter is used.

## 5. Conclusions

We propose an affordable and reliable methodology to measure the color in samples that do not fully cover the aperture of a spectrophotometer. We show that, following colorimetric and statistical criteria, the only setting providing indistinguishable colors from the real one (measured with no adaptor) is the 50%-reducing adaptor on the 3-mm aperture (SAV). In addition, statistical analyses suggest the choice of a white adaptor. Our research demonstrates that, by using the proposed methodology, it is possible to gather ecological information in a sound, inexpensive, and non-destructive way from the color of leaves, petals, and other small samples.

## Figures and Tables

**Table 1:** Data of the six herbarium samples belonging to the Campanulaceae family and used in this study.

Herbarium Code ^1.^	Species	Location ^2^	Collector	Year
SANT 210	*Campanula latifolia* L.	ES. Peña Oroel, Jaca	Bellot, Vieitez	1947
SANT 232	*Campanula trachelium* L.	ES. Province of Barcelona	Marcos	1944
SANT 21287	*Campanula adsurgens* Levier & Leresche	ES. Triacastela, Lugo	J. Amigo, J. Giménez de Azcárate	1990
SANT 29493	*Campanula versicolor* Andrews	IT. Santa Cesarea, Lecce	J. Izco	1994
SANT 51406	*Campanula glomerata* L.	ES. Penedos de Oulego, Ourense	J. Amigo	2004
SANT 62512	*Campanula mollis* L.	ES. Sierra de Grazalema, Cádiz	J. Izco	1976

^1^ SANT: Herbarium of the University of Santiago de Compostela; ^2^ ES: Spain; IT: Italy.

**Table 2:** Colorimetric analysis (total color differences, ΔE*_ab_) of the measurements with black 50%, white 50%, black 70% and white 70% adaptors, compared to those obtained with no adaptor. ΔE*_ab_ below the threshold of perception (< 3.0 CIELAB units) are in bold and underlined. Plants marked with an asterisk (*) are dry material from herbarium specimens.

		***J. montana***	***T. caeruleum***	***C. rotundifolia***	***C. isophylla***	***H. hederaceus***	***J. laevis***
**MAV**	50% black	9.5	7.4	4.5	3.8	5.6	5.3
	50% white	25.0	26.1	24.1	24.5	22.8	23.9
	70% black	22.1	19.1	15.0	12.4	17.4	12.3
	70% white	46.4	48.3	46.8	47.8	45.1	47.1
**SAV**	50% black	**2.5**	**2.4**	**2.0**	**0.8**	**0.7**	**1.2**
	50% white	**2.1**	**2.7**	**1.8**	**1.1**	**1.1**	**1.4**
	70% black	21.1	19.6	15.9	11.7	16.1	11.0
	70% white	34.4	36.1	34.4	35.9	34.6	34.9
		C. adsurgens *	C. latifolia *	C. mollis *	C. glomerate *	C. trachelium *	C. vesicular *
**MAV**	50% black	7.4	8.5	4.1	6.5	9.7	5.4
	50% white	14.8	18.6	15.9	20.4	17.1	19.1
	70% black	19.9	18.1	17.4	21.0	21.1	19.8
	70% white	34.5	40.1	35.2	41.8	37.5	39.0
**SAV**	50% black	**2.1**	**2.9**	**1.5**	**2.4**	**2.9**	**2.1**
	50% white	**1.1**	**2.4**	**2.8**	**2.4**	**2.0**	**0.7**
	70% black	18.9	17.6	17.3	18.4	19.5	18.9
	70% white	30.6	35.1	26.4	37.2	34.0	33.4
		**Green**	**Foliage**	**Yellow Green**	**Bluish Green**		
**MAV**	50% black	10.7	6.6	12.7	7.9		
	50% white	27.1	22.3	26.3	13.1		
	70% black	34.5	20.4	42.4	29.7		
	70% white	54.2	45.6	57.2	31.4		
**SAV**	50% black	**2.8**	**1.9**	**2.9**	**2.5**		
	50% white	**1.3**	**1.4**	**2.6**	**0.5**		
	70% black	29.7	16.5	36.6	23.9		
	70% white	47.2	38.7	50.9	25.2		

**Table 3:** Results of the k-means cluster analysis on SAV measurements with the different adaptors compared to no adaptor. The number of clusters formed (*k*) and the related approximately unbiased (AU) p-values are indicated for each k-cluster. Clusters with AU *p*-value equal to or greater than 95% are considered strongly supported by data. The clusters where the SAV measurements with no adaptor (real color) fall are indicated in bold.

Fresh Material				Herbarium Material			
		AU	k			AU	k
		*p*-Value				*p*-Value	
*Jasione laevis*	**A = {100% SAV, 100% SAV50W, 100% SAV50B, 100% SAV70W}**	**97**	2	*Campanula adsurgens*	**A = {100% SAV, 100% SAV50W, 92% SAV50B}**	**99**	3
	B = {100% SAV70B}	97			B = {100% SAV70W, 8% SAV70B}	97	
					C = {10% SAV70B}	97	
*Jasione montana*	**A = {68% SAV, 100% SAV50W, 100% SAV50B}**	**86**	4	*Campanula glomerata*	**A = {100% SAV, 96% SAV50W, 96% SAV50B}**	**89**	3
	**B = {32% SAV}**	**100**			B = {100% SAV70W, 4% SAV50W}	97	
	C = {100% SAV70W}	100			C = {100% SAV70B, 4% SAV50B}	99	
	D = {100% SAV70B}	100					
*Campanula isophylla*	**A = {100% SAV, 100% SAV50W, 100% SAV50B, 100% SAV70W}**	**97**	2	*Campanula latifolia*	**A = {100% SAV, 100% SAV50W, 92% SAV50B}**	**99**	3
	B = {100% SAV70B}	97			B = {100% SAV70W, 8% SAV50B}	97	
					C = {100% SAV70B}	97	
*Campanula rotundifolia*	**A = {56% SAV, 56% SAV50B, 60% SAV50W}**	**88**	4	*Campanula mollis*	**A = {84% SAV, 72% SAV50W, 88% SAV50B}**	**89**	3
	**B = {44% SAV, 44% SAV50B, 40% SAV50W}**	**88**			**B = {16%SAV, 28% SAV50W, 12% SAV50B}**	**86**	
	C = {100% SAV70B}	100			C = {100% SAV70B}	99	
	D = {100% SAV70W}	100					
*Trachelium caeruleum*	**A = {100% SAV, 100% SAV50W, 100% SAV50B}**	**100**	3	*Campanula trachelium*	**A = {100% SAV, 100% SAV50W, 100% SAV50B}**	**100**	3
	B = {100% SAV70W}	97			B= {100% SAV70W}	99	
	C= {100% SAV70B}	97			C= {100% SAV70B}	97	
*Hesperocodon hederaceus*	**A = {100% SAV, 100% SAV50B, 100% SAV50W}**	**100**	3	*Campanula versicolor*	**A = {100% SAV, 100% SAV50W, 100% SAV50B}**	**99**	3
	B = {100% SAV70B}	100			B = {100% SAV70W}	97	
	C = {100% SAV70W}	100			C = {100% SAV70B}	97	

## Appendix A

**Table A1:** Partial (∆L*, ∆a*, ∆b*) and total (∆E*_ab_) color differences in absolute values with black 50%, white 50%, black 70%, and white 70% adaptors compared to those obtained with no adaptor.

		***Jasione montana***		***Trachelium caeruleum***		***Campanula rotundifolia***		***Campanula isophylla***		***Hesperocodon hederacerus***		***Jasione laevis***	
**MAV**	50% black	ΔL*: 3.4 Δa*: 4.5 Δb*: 7.6	ΔE*_ab_: 9.5	ΔL*: 2.2 Δa*: 3.7 Δb*: 6.1	ΔE*_ab_: 7.4	ΔL*: 0.9 Δa*: 2.6 Δb*: 3.5	ΔE*_ab_: 4.5	ΔL*: 0.6 Δa*: 2.5 Δb*: 2.8	ΔE*_ab_: 3.8	ΔL*: 2.4 Δa*: 2.7 Δb*: 4.3	ΔE*_ab_: 5.6	ΔL*: 1.9 Δa*: 2.9 Δb*: 3.9	ΔE*_ab_: 5.3
	50% white	ΔL*: 17.3 Δa*: 9.7 Δb*: 15.2	ΔE*_ab_: 25.0	ΔL*: 20.6 Δa*: 9.4 Δb*: 13.0	ΔE*_ab_: 26.1	ΔL*: 20.4 Δa*: 8.4 Δb*: 9.6	ΔE*_ab_: 24.1	ΔL*: 22.3 Δa*: 7.5 Δb*: 6.9	ΔE*_ab_: 24.5	ΔL*: 18.5 Δa*: 8.2 Δb*: 10.4	ΔE*_ab_: 22.8	ΔL*: 22.0 Δa*: 6.9 Δb*: 6.5	ΔE*_ab_: 23.9
	70% black	ΔL*: 11.8 Δa*: 9.6 Δb*: 16.1	ΔE*_ab_: 22.1	ΔL*: 8.7 Δa*: 9.4 Δb*: 14.2	ΔE*_ab_: 19.1	ΔL*: 7.4 Δa*: 7.7 Δb*: 10.5	ΔE*_ab_: 15.0	ΔL*: 5.9 Δa*: 7.0 Δb*: 8.3	ΔE*_ab_: 12.4	ΔL*: 9.8 Δa*: 7.9 Δb*: 12.0	ΔE*_ab_: 17.4	ΔL*: 6.5 Δa*: 6.6 Δb*: 8.1	ΔE*_ab_: 12.3
	70% white	ΔL*: 37.0 Δa*: 14.8 Δb*: 23.8	ΔE*_ab_: 46.4	ΔL*: 41.5 Δa*: 13.9 Δb*: 20.5	ΔE*_ab_: 48.3	ΔL*: 42.3 Δa*: 12.0 Δb*: 15.8	ΔE*_ab_: 46.8	ΔL*: 45.1 Δa*: 10.5 Δb*: 12.1	ΔE*_ab_: 47.8	ΔL*: 39.6 Δa*: 12.4 Δb*: 17.8	ΔE*_ab_: 45.1	ΔL*: 44.9 Δa*: 9.2 Δb*: 10.8	ΔE*_ab_: 47.1
**SAV**	50% black	ΔL*: 1.7 Δa*: 0.6 Δb*: 1.8	ΔE*_ab_: 2.5	ΔL*: 1.2 Δa*: 0.8 Δb*: 1.9	ΔE*_ab_ 2.4	ΔL*: 1.2 Δa*: 0.7 Δb*: 1.4	ΔE*_ab_: 2.0	ΔL*: 0.1 Δa*: 0.5 Δb*: 0.7	ΔE*_ab_: 0.8	ΔL*: 0.0 Δa*: 0.2 Δb*: 0.6	ΔE*_ab_: 0.7	ΔL*: 0.1 Δa*: 0.7 Δb*: 1.0	ΔE*_ab_: 1.2
	50% white	ΔL*: 0.9 Δa*: 0.9 Δb*: 1.7	ΔE*_ab_: 2.1	ΔL*: 0.7 Δa*: 1.2 Δb*: 2.3	ΔE*_ab_: 2.7	ΔL*: 0.6 Δa*: 1.0 Δb*: 1.3	ΔE*_ab_: 1.8	ΔL*: 0.6 Δa*: 0.7 Δb*: 0.6	ΔE*_ab_: 1.1	ΔL*: 0.9 Δa*: 0.3 Δb*: 0.5	ΔE*_ab_: 1.1	ΔL*: 0.6 Δa*: 0.9 Δb*: 1.0	ΔE*_ab_: 1.4
	70% black	ΔL*: 9.4 Δa*: 9.3 Δb*: 16.5	ΔE*_ab_: 21.1	ΔL*: 6.0 Δa*: 10.0 Δb*: 15.8	ΔE*_ab_: 19.6	ΔL*: 6.9 Δa*: 8.3 Δb*: 11.6	ΔE*_ab_: 15.9	ΔL*: 3.9 Δa*: 7.3 Δb*: 8.3	ΔE*_ab_: 11.7	ΔL*: 7.3 Δa*: 8.0 Δb*: 12.0	ΔE*_ab_: 16.1	ΔL*: 4.2 Δa*: 6.6 Δb*: 7.7	ΔE*_ab_: 11.0
	70% white	ΔL*: 24.6 Δa*: 11.7 Δb*: 21.0	ΔE*_ab_: 34.4	ΔL*: 27.6 Δa*: 12.1 Δb*: 19.8	ΔE*_ab_: 36.1	ΔL*: 29.5 Δa*: 10.8 Δb*: 14.1	ΔE*_ab_: 34.4	ΔL*: 33.1 Δa*: 9.4 Δb*: 10.1	ΔE*_ab_: 35.9	ΔL*: 29.2 Δa*: 10.7 Δb*: 15.1	ΔE*_ab_: 34.6	ΔL*: 32.7 Δa*: 8.2 Δb*: 9.0	ΔE*_ab_: 34.9
		***Campanula adsurgens* ***		***Campanula latifolia* ***		***Campanula mollis* ***		***Campanula glomerata* ***		***Campanula trachelium* ***		***Campanula vesicular* ***	
**MAV**	50% black	ΔL*: 6.2 Δa*: 0.4 Δb*: 4.1	ΔE*_ab_: 7.4	ΔL*: 6.6 Δa*: 0.8 Δb*: 5.4	ΔE*_ab_: 8.5	ΔL*: 0.8 Δa*: 0.4 Δb*: 4.0	ΔE*_ab_: 4.1	ΔL*: 3.3 Δa*: 2.0 Δb*: 5.2	ΔE*_ab_: 6.5	ΔL*: 5.3 Δa*: 1.6 Δb*: 7.9	ΔE*_ab_: 9.7	ΔL*: 3.0 Δa*: 0.6 Δb*: 4.5	ΔE*_ab_: 5.4
	50% white	ΔL*: 11.3 Δa*: 0.8 Δb*: 9.5	ΔE*_ab_: 14.8	ΔL*: 15.4 Δa*: 0.9 Δb*: 10.3	ΔE*_ab_: 18.6	ΔL*: 14.8 Δa*: 0.4 Δb*: 5.8	ΔE*_ab_: 15.9	ΔL*: 15.0 Δa*: 5.4 Δb*: 12.7	ΔE*_ab_: 20.4	ΔL*: 13.3 Δa*: 2.1 Δb*: 10.5	ΔE*_ab_: 17.1	ΔL*: 15.8 Δa*: 0.5 Δb*: 10.7	ΔE*_ab_: 19.1
	70% black	ΔL*: 16.5 Δa*: 0.4 Δb*: 11.1	ΔE*_ab_: 19.9	ΔL*: 13.4 Δa*: 1.5 Δb*: 12.0	ΔE*_ab_: 18.1	ΔL*: 15.5 Δa*: 0.2 Δb*: 8.0	ΔE*_ab_: 17.4	ΔL*: 14.1 Δa*: 5.3 Δb*: 14.6	ΔE*_ab_: 21.0	ΔL*: 16.6 Δa*: 2.9 Δb*: 12.7	ΔE*_ab_: 21.1	ΔL*: 14.3 Δa*: 0.9 Δb*: 13.7	ΔE*_ab_: 19.8
	70% white	ΔL*: 29.2 Δa*: 0.9 Δb*: 18.3	ΔE*_ab_: 34.5	ΔL*: 35.8 Δa*: 1.3 Δb*: 18.1	ΔE*_ab_: 40.1	ΔL*: 32.8 Δa*: 0.9 Δb*: 12.8	ΔE*_ab_: 35.2	ΔL*: 33.9 Δa*: 8.8 Δb*: 22.7	ΔE*_ab_: 41.8	ΔL*: 32.2 Δa*: 3.3 Δb*: 19.0	ΔE*_ab_: 37.5	ΔL*: 33.2 Δa*: 0.2 Δb*: 20.6	ΔE*_ab_: 39.0
**SAV**	50% black	ΔL*: 0.0 Δa*: 0.1 Δb*: 2.1	ΔE*_ab_: 2.1	ΔL*: 2.3 Δa*: 0.2 Δb*: 1.8	ΔE*_ab_: 2.9	ΔL*: 0.8 Δa*: 0.2 Δb*: 1.2	ΔE*_ab_: 1.5	ΔL*: 1.6 Δa*: 0.6 Δb*: 1.8	ΔE*_ab_: 2.4	ΔL*: 2.5 Δa*: 0.5 Δb*: 1.3	ΔE*_ab_: 2.9	ΔL*: 1.7 Δa*: 0.2 Δb*: 1.2	ΔE*_ab_: 2.1
	50% white	ΔL*: 0.2 Δa*: 0.6 Δb*: 0.9	ΔE*_ab_: 1.1	ΔL*: 2.0 Δa*: 0.0 Δb*: 1.5	ΔE*_ab_: 2.4	ΔL*: 2.3 Δa*: 0.2 Δb*: 1.6	ΔE*_ab_: 2.8	ΔL*: 0.1 Δa*: 0.9 Δb*: 2.2	ΔE*_ab_: 2.4	ΔL*: 1.2 Δa*: 0.3 Δb*: 1.6	ΔE*_ab_: 2.0	ΔL*: 0.3 Δa*: 0.2 Δb*: 0.6	ΔE*_ab_: 0.7
	70% black	ΔL*: 14.9 Δa*: 0.1 Δb*: 11.6	ΔE*_ab_: 18.9	ΔL*: 12.8 Δa*: 1.5 Δb*: 11.9	ΔE*_ab_: 17.6	ΔL*: 16.3 Δa*: 0.2 Δb*: 5.8	ΔE*_ab_: 17.3	ΔL*: 10.9 Δa*: 4.6 Δb*: 14.1	ΔE*_ab_: 18.4	ΔL*: 13.9 Δa*: 3.0 Δb*: 13.4	ΔE*_ab_: 19.5	ΔL*: 13.7 Δa*: 0.6 Δb*: 13.0	ΔE*_ab_: 18.9
	70% white	ΔL*: 25.1 Δa*: 0.6 Δb*: 17.5	ΔE*_ab_: 30.6	ΔL*: 30.4 Δa*: 1.6 Δb*: 17.5	ΔE*_ab_: 35.1	ΔL*: 24.2 Δa*: 0.4 Δb*: 10.6	ΔE*_ab_: 26.4	ΔL*: 29.6 Δa*: 7.2 Δb*: 21.4	ΔE*_ab_: 37.2	ΔL*: 28.0 Δa*: 3.5 Δb*: 19.1	ΔE*_ab_: 34.0	ΔL*: 27.4 Δa*: 0.2 Δb*: 19.1	ΔE*_ab_: 33.4
			**Green**			**Foliage**		**Yellow Green**			**Bluish Green**		
**MAV**	50% black		ΔL*: 4.6 Δa*: 7.1 Δb*: 6.5	ΔE*_ab_: 10.7		ΔL*: 2.5 Δa*: 3.3 Δb*: 5.2	ΔE*_ab_: 6.6	ΔL*: 7.2 Δa*: 3.6 Δb*: 9.8		ΔE*_ab_: 12.7	ΔL*: 6.6 Δa*: 4.4 Δb*: 0.6	ΔE*_ab_: 7.9	
	50% white		ΔL*: 11.3 Δa*: 19.3 Δb*: 15.4	ΔE*_ab_: 27.1		ΔL*: 17.1 Δa*: 8.9 Δb*: 11.3	ΔE*_ab_: 22.3	ΔL*: 4.9 Δa*: 9.9 Δb*: 23.9		ΔE*_ab_: 26.3	ΔL*: 5.7 Δa*: 11.8 Δb*: 0.5	ΔE*_ab_: 13.1	
	70% black		ΔL*: 17.9 Δa*: 22.3 Δb*: 19.4	ΔE*_ab_: 34.5		ΔL*: 11.3 Δa*: 9.6 Δb*: 14.1	ΔE*_ab_: 20.4	ΔL*: 26.1 Δa*: 13.0 Δb*: 30.7		ΔE*_ab_: 42.4	ΔL*: 25.4 Δa*: 15.4 Δb*: 1.1	ΔE*_ab_: 29.7	
	70% white		ΔL*: 27.7 Δa*: 36.0 Δb*: 29.7	ΔE*_ab_: 54.2		ΔL*: 38.3 Δa*: 14.5 Δb*: 20.0	ΔE*_ab_: 45.6	ΔL*: 13.5 Δa*: 23.8 Δb*: 50.2		ΔE*_ab_: 57.2	ΔL*: 15.3 Δa*: 27.4 Δb*: 1.2	ΔE*_ab_: 31.4	
**SAV**	50% black		ΔL*: 1.6 Δa*: 1.6 Δb*: 1.6	ΔE*_ab_: 2.8		ΔL*: 1.1 Δa*: 0.8 Δb*: 1.3	ΔE*_ab_: 1.9	ΔL*: 2.0 Δa*: 0.7 Δb*: 1.9		ΔE*_ab_: 2.9	ΔL*: 2.3 Δa*: 0.9 Δb*: 0.0	ΔE*_ab_: 2.5	
	50% white		ΔL*: 0.0 Δa*: 1.0 Δb*: 0.9	ΔE*_ab_: 1.3		ΔL*: 0.3 Δa*: 0.8 Δb*: 1.2	ΔE*_ab_: 1.4	ΔL*: 1.3 Δa*: 0.6 Δb*: 2.2		ΔE*_ab_: 2.6	ΔL*: 0.3 Δa*: 0.3 Δb*: 0.1	ΔE*_ab_: 0.5	
	70% black		ΔL*: 12.8 Δa*: 20.3 Δb*: 17.4	ΔE*_ab_: 29.7		ΔL*: 6.4 Δa*: 8.7 Δb*: 12.5	ΔE*_ab_: 16.5	ΔL*: 20.3 Δa*: 11.8 Δb*: 28.1		ΔE*_ab_: 36.6	ΔL*: 19.6 Δa*: 13.7 Δb*: 0.2	ΔE*_ab_: 23.9	
	70% white		ΔL*: 21.1 Δa*: 31.3 Δb*: 28.4	ΔE*_ab_: 47.2		ΔL*: 29.9 Δa*: 13.2 Δb*: 20.7	ΔE*_ab_: 38.7	ΔL*: 9.3 Δa*: 20.0 Δb*: 45.8		ΔE*_ab_: 50.9	ΔL*: 11.3 Δa*: 22.4 Δb*: 2.0	ΔE*_ab_: 25.2	
